# Isolation of serotype-specific antibodies against dengue virus non-structural protein 1 using phage display and application in a multiplexed serotyping assay

**DOI:** 10.1371/journal.pone.0180669

**Published:** 2017-07-06

**Authors:** Kebaneilwe Lebani, Martina L. Jones, Daniel Watterson, Andrea Ranzoni, Renee J. Traves, Paul R. Young, Stephen M. Mahler

**Affiliations:** 1 Australian Institute for Bioengineering and Nanotechnology, The University of Queensland, Brisbane, Queensland, Australia; 2 ARC Training Centre for Biopharmaceutical Innovation, The University of Queensland, Brisbane, Queensland, Australia; 3 School of Chemistry and Molecular Biosciences, The University of Queensland, Brisbane, Queensland, Australia; 4 Australian Infectious Diseases Research Centre, The University of Queensland, Brisbane, Queensland, Australia; 5 Institute for Molecular Bioscience, The University of Queensland, Brisbane, Queensland, Australia; Univ of Mass Med Schl, UNITED STATES

## Abstract

The multidimensional nature of dengue virus (DENV) infections, which can be caused by four distinct serotypes of the virus, complicates the sensitivity of assays designed for the diagnosis of infection. Different viral markers can be optimally detected at different stages of infection. Of particular clinical importance is the early identification of infection, which is pivotal for disease management and the development of blood screening assays. Non-structural protein 1 (NS1) is an early surrogate marker of infection and its detection in serum coincides with detectable viraemia. The aim of this work was to isolate and characterise serotype-specific monoclonal antibodies that bind to NS1 for each of the four DENV serotypes. This was achieved using phage display and a subtractive biopanning strategy to direct the antibody selection towards serotype-specific epitopes. This antibody isolation strategy has advantages over immunisation techniques where it is difficult to avoid antibody responses to cross-reactive, immunodominant epitopes. Serotype specificity to recombinant antigen for each of the antibodies was confirmed by Enzyme Linked Immunosorbent Assay (ELISA) and Surface Plasmon Resonance. Confirmation of binding to native DENV NS1 was achieved using ELISA and immunofluorescence assay on DENV infected Vero cells. No cross-reactivity with Zika or Kunjin viruses was observed. A previously isolated pan-reactive antibody that binds to an immunodominant epitope was able to pair with each of the serotype-specific antibodies in a sandwich ELISA, indicating that the serotype specific antibodies bind to epitopes which are all spatially distinct from the immunodominant epitope. These antibodies were suitable for use in a multiplexed assay for simultaneous detection and serotyping of DENV NS1 in human serum. This work demonstrates that phage display coupled with novel biopanning strategies is a valuable *in vitro* methodology for isolation of binders that can discern amongst antigens with high homology for diagnostic applicability.

## Introduction

Dengue virus infections are a significant public health burden. Dengue virus belongs to the genus flavivirus and is transmitted by the mosquito vectors *Aedes aegypti* and *Aedes albopictus*. Four distinct serotypes of DENV have been identified and often co-circulate together or with other flaviviruses such as Yellow Fever Virus, Japanese Encephalitis Virus, West Nile Virus and the emerging Zika virus (ZIKV) [1, 2]. 2010 population data and cartographic methods estimated the global dengue infection rate at 390 million infections annually, 96 million of which were symptomatic [3]. Hyper-endemic areas in the tropics and sub-tropics particularly struggle with timely diagnosis of acute infection as well as epidemiological surveillance of the infective flavivirus and/or the infective dengue serotype.

The dengue virus is a single-stranded, positive sense, RNA virus with a 11 kb genome which encodes a polyprotein which is post-translationally cleaved to yield 3 structural proteins (capsid, membrane and envelope) and 7 non-structural proteins (NS1, NS2A, NS2B, NS3, NS4A, NS4B and NS5) [4].The structure, assembly and pathogenesis of the dengue virus allow different options for diagnosis.

Diagnosis of dengue infections can be achieved by several means, including virus culture, nucleic acid tests, IgG and IgM serology tests and antigen detection tests such as those that detect NS1 [5]. Each of these tests has its advantages and limitations which have been well reviewed [6, 7].

This study specifically investigates the detection of NS1, a multi-functional glycoprotein that is both essential for viral replication and a key moderator of host innate immune responses [8, 9]. NS1 has been established as a good surrogate marker for infection with a direct correlation to disease severity [10, 11]. The NS1 glycoprotein is not associated with the virion and is found within the infected cell as a membrane associated dimer of about 90 kDa [12]. NS1 is also secreted as a lipid-associated 310 kDa hexamer, made up of a trimer of dimers and lipid cargo [13, 14]. It is this soluble hexamer that is detected in the serum for differential diagnosis from other febrile diseases. Discernment of high sequence homology between dengue virus NS1 and NS1 from other flaviviruses requires highly specific antibodies that target dengue-specific regions on NS1, as individuals in endemic regions can be subject to multiple, sequential infections with DENV serotypes or other co-circulating flaviviruses, such as Yellow Fever and Zika. The ability to discern amongst serotypes of DENV requires an even higher level of specificity.

Our aim was to isolate antibodies which could discern between the four serotypes of DENV, which could potentially be used to improve the utility of DENV NS1 diagnostic ELISA, by adding a serotyping capability. We interrogated antibody phage libraries using a subtractive biopanning strategy to direct the selection of binders towards unique epitopes of each of the four serotypes of DENV NS1. Four antibody fragments were isolated, each specifically binding NS1 from a different dengue virus serotype. These antibody fragments were then characterised as fully assembled, human IgG1 antibodies. Interestingly, the epitopes for the four isolated mAbs do not appear to overlap with the immunodominant, cross reactive 'wing domain' epitope identified by Akey et al [15] and can therefore be paired with a single pan-reactive mAb against this epitope in a sandwich ELISA.

## Methods

### Recombinant dengue virus NS1

Recombinant NS1 (rNS1) from each DENV serotype expressed in CHO cells, from Alere Inc (San Diego), were gifted from Prof Matthew Cooper (Institute for Molecular Bioscience, The University of Queensland).

### Pan-reactive anti-DENV NS1 antibody

Gus2 mAb was derived from a mouse hybridoma as previously described, after immunisation of BALB/C mice with purified soluble DENV-2 NS1 protein [16].

### Phage displayed naive libraries

Two human naïve phage display libraries containing antibody fragments as fusions to the head capsid protein gIIIp of bacteriophage M13 were used. The 'Sheets' library [17], containing scFv fragments in pHEN1 phagemid, was obtained from Prof James Marks (University of California San Francisco) and a Fab antibody library in pCES1 phagemid, was obtained from the Australian Red Cross Blood Service, who created the library using methods described in [18]. The ‘Sheets’ library used in this work has a documented diversity of 6.7 x 10^9^ independent scFv clones [17] and the Fab library has a diversity of 1.0 x 10^10^ independent clones (personal communication).

### Biopanning

**Fig 1:**
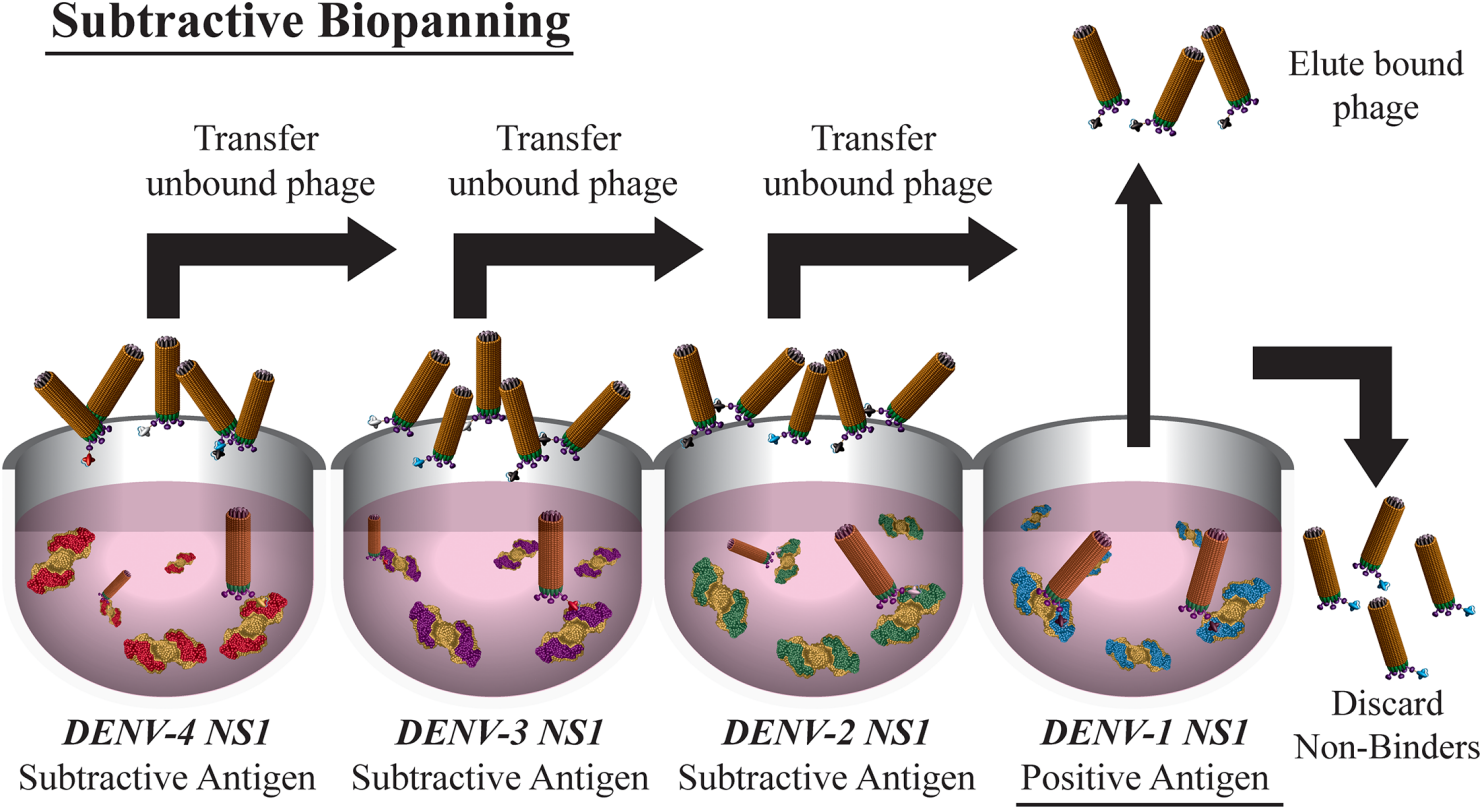
General overview of subtractive biopanning of a phage library displaying antibody fragments. Recombinant antigen from all four different DENV serotypes was adsorbed on polystyrene immunotubes. The phage library was exposed to each tube in turn. The first three tubes were used for subtractive purposes to deplete binders against homologous regions amongst the rNS1. Only binders in the last tube against the target antigen were eluted for further characterisation.

Antigen binders with desired properties were enriched by several cycles of phage display-driven selection (biopanning) guided by standard methods [19]. To isolate serotype-specific binders a subtractive biopanning strategy was employed to isolate binders to immobilised rNS1 from each serotype, outlined in Fig 1. For example, in instances where binders to DENV-1 rNS1 were sought, the phage library was first added to an immunotube coated with DENV-4 rNS1. Unbound phage were decanted from that immunotube and transferred to another immunotube coated with DENV-3 rNS1 and then to a DENV-2 rNS1 coated immunotube, before finally being exposed to DENV-1 rNS1 coated on the last immunotube. In the final step, the unbound non-specific phage particles were discarded while the bound phage particles which are specific for the antigen from the target serotype were eluted with a low pH buffer. Due to a shortage of DENV-1 rNS1, this was not included in the subtractive rounds when isolating binders to the other three serotypes, where the order of subtraction was: for DENV-2, subtraction with DENV-3 followed by DENV-4 rNS1; for DENV-3, subtraction with DENV-2 followed by DENV-4; and for DENV-4, subtraction with DENV-2 followed by DENV-3. The first round of biopanning was carried out with 50 μg/mL of rNS1 immobilized on Maxisorp Immunotubes (Nunc). The amplified phage particles were enriched by at least two more iterative rounds of biopanning where 10 μg/mL of rNS1 was immobilized to immunotubes.

### Direct Enzyme Linked Immunosorbent Assay (ELISA)

To determine the binding specificity of isolated antibody fragments displayed on phage particles or of whole antibodies, direct binding ELISA against DENV rNS1 was performed. 200 μL of purified rNS1 from DENV1-4 at 3 μg/mL in phosphate buffered saline (PBS) was coated on a MaxiSorp plate (NUNC) overnight at 4°C. The unbound protein was discarded and followed by 3 consecutive washes with PBS containing 0.1% Tween20 (PBS-T). The plate was blocked using PBS with 2.5% milk powder (M-PBS). The blocking buffer was discarded and 200 μL of 3 μg/mL antibody or phage diluted in the M-PBS blocking buffer was added to relevant wells. Following one hour incubation and three washes with PBS-T, the plate was probed with 200 μL of HRP-conjugated anti-M13 (GE Healthcare) or HRP-conjugated goat anti mouse [H+L] IgG (Life Technologies) or HRP-conjugated goat anti human IgG [γ chain specific] (Sigma) diluted to 0.1 μg/mL in PBS with casein. Three final washes with PBS-T were performed then 100 μL TMB substrate (Sigma Aldrich) was added to develop the chromogenic signal. The reaction was stopped with 2 N sulphuric acid and the absorbance at 450 nm was measured using the Spectramax microplate reader (Molecular Devices).

### Reformatting of antibody fragments

Unique, antigen specific antibody fragments from the scFv and Fab libraries were reformatted to fully human IgG1 using In-Fusion^®^ cloning (Clontech) of the variable light (kappa or lambda) and heavy chains into antibody expression vectors containing the relevant human constant region sequences [20].

### Expression and purification of IgG1

The vectors expressing the introduced variable regions were transfected into CHO-S cells (ThermoFisher Scientific) using PEI-pro (Polyplus transfection) and maintained using standard methods [21]. The expressed antibodies were secreted into the medium enabling harvesting by centrifugation followed by double filtration with Sartobran P300, 0.45/0.2 μm capsule filters (Sartorium Stedim Biotech) on day 10 post-transfection. The antibodies were purified on the AKTA Explorer chromatography system (GE Healthcare) using a 5 mL HiTrap MabSelect SuRe column (GE Healthcare). The antibodies were eluted using 0.1 M glycine pH 2.7 and neutralized with 1/10 total elution volume of 1 M Tris pH 7.4. The neutralized antibodies were desalted and concentrated using Macrosep Advance Centrifugal Devices (Pall).

### Vero cell infection

Vero (African green monkey kidney) cells (ATCC) were adapted and grown using Opti-MEM (Gibco) supplemented with 3% fetal calf serum (Bovogen) in T25 vented flasks (Greiner Bio-One) in a humidified, 37°C incubator with 5% C0_2_. Cells were split using 0.5% Trypsin versene (Gibco) and seeded at 50% confluence in either T25 vented flasks or in 96-well cell culture plates (Corning^®^ Costar^®^). Cells were infected with DENV-1, DENV-2, DENV-3, DENV-4 (all East Timor strains corresponding to GenBank entries 440432.1, 440433.1, 440434.1 and 440435.1 respectively) or Kunjin virus (KUNV, a highly attenuated, Australasian variant of West Nile virus) at 0.1 multiplicity of infection in serum-free Opti-MEM for 2 hours at 37°C. After infection, the infection medium was replaced with Opti-MEM supplemented with 2% fetal calf serum. DENV infected cells were cultured for three days while KUNV infected cells were cultured for two days post infection. Cell culture supernatants from T25 flasks were harvested and concentrated (Macrosep Advance Centrifugal Devices, Pall) then used in a sandwich ELISA to detect secreted NS1 while infected cells in 96-well cell culture plates were fixed and used in immunofluorescence assays.

### Immunofluorescence against native NS1

Immunofluorescence assays were performed on cells fixed with paraformaldehyde and permeabilised with 0.1% Triton X100. Wells were blocked with 2% casein in PBS-T. Serotype-specific antibodies against DENV NS1 were used at a concentration of 10 μg/mL in blocking solution, while a mouse antibody against the flaviviral envelope protein was used at a concentration of 1 μg/mL also in blocking solution. Following one hour incubation, three washes were performed using PBS-T. Anti-mouse Alexa Fluor 546 (Life Technologies), anti-human Alexa Fluor 488 (Jackson ImmunoResearch) and Hoescht (Thermo Scientific) in blocking buffer were added to the wells and incubated for a further hour before another 3 washes with PBS-T. Immuno-fluorescence imaging was performed at 40 x magnification using the InCell Analyzer (GE Healthcare).

### Surface Plasmon Resonance (SPR)

Antigen—antibody interaction kinetics and affinity were determined by SPR using the Biacore T200 (GE Healthcare). Approximately 9,000 resonance units (RU) of anti-human Fc (GE Healthcare, Human Antibody Capture Kit) or anti-mouse Fc (GE Healthcare, Mouse Antibody Capture Kit) was immobilized on CM5 sensor chips. Human-derived serotype-specific or mouse-derived pan-reactive Gus2 Anti-DENV NS1 antibodies were captured onto the relevant Fc-capture flow cell, while another uncoated flow cell acted as the reference surface for each Fc-capture. Two-fold, serial dilutions of recombinant NS1 were made from 1 μM to 62.5 nM using the instrument running buffer (1 X HBS EP^+^) with NaCl added to 300 mM final concentration. High Performance Multi-Cycle Kinetics was used for the serotype-specific mAbs, while Single Cycle Kinetics was used for Gus2 (Biacore T200 protocols). The flow rate used for injection of the capture antibody and the analyte was 30 μL/min. Variable anti-DENV NS1 capture times were used to allow approximately 140 RU of capture, then association with different concentrations of analyte was allowed to proceed for 3 min. Dissociation of the antigen from the antibody was measured over 15 min. Regeneration of the anti-human Fc surface with 3 M MgCl_2_, or the anti-mouse Fc surface with 10mM glycine, pH 1.7, was performed at 10 μL/min for 30 sec after each capture and analyte binding cycle. The evaluation software generated sensorgrams of the antigen-antibody interactions yielding association (k_a_) and dissociation (k_d_) rate constants from which the equilibrium dissociation constant (K_D_) was calculated assuming a 1:1 model of interaction.

### Sandwich ELISA—Binding to native viral antigen

The binding specificity of the candidate monoclonal antibodies (mAbs) for native viral antigen derived from DENV1-4 and the related flavivirus Zika (ZIKV) was examined by sandwich ELISA. A cocktail of broadly flavi-reactive murine antibodies were coated on a MaxiSorp plate (NUNC) at 2 μg/mL in bicarbonate buffer pH 9.6 overnight at 4°C. Unbound mAbs were removed and a milk diluent blocking solution (KPL) was applied for 1 hr at room temperature. The blocking solution was removed and native DENV1-4 and ZIKV, in the form of virus-infected Vero cell supernatant, was diluted in blocking solution and applied at 50 μL per well. Uninfected Vero cell supernatant was included as a negative control. The NS1 capture was allowed to occur for 1 hr at 37°C before washing with PBS-T to remove unbound antigen. Candidate human mAbs (9H2, 4C11, 7G11 and 6A5) along with a biotinylated flavi-specific NS1 antibody cocktail were used for detection at 2 μg/mL in blocking solution for 1 hr at 37°C. The plate was washed with PBS-T, before the addition of HRP-conjugated goat anti-human antibody (1:2000 dilution) in blocking solution for the human monoclonals and streptavidin-HRP (1:2000 dilution) for the flavi-specific cocktail. Following a 1hr incubation at 37°C plates were washed and the addition of 50 μL per well of TMB substrate allowed visualization of successful capture and detection of viral NS1 by chromogenic colour reaction. Colour development was ceased by the addition of 1M sulphuric acid, with a microtitre reader implemented to quantify the absorbance at 450nm. Absorbances from the control supernatant wells were subtracted from the test samples for final analysis.

### Sandwich ELISA—Compatibility with pan-specific DENV mAb Gus2

To investigate use of the serotype-specific antibodies in a sandwich ELISA format each of the four serotype-specific mAbs were tested for compatibility with Gus2, a broadly cross-reactive DENV specific murine monoclonal. Firstly the new serotype-specific antibodies were tested for binding to a peptide corresponding to residues 111–125 of NS1 (HK**YSWK**S**WGKAK**IIG–conserved residues across all four serotypes in bold), which contains the highly conserved, immunodominant 'wing domain' linear epitope of NS1 [15], and known to be the binding epitope of Gus2 antibody [22]. An unrelated peptide (SKGTPMYSVDL) was used as a negative control. Both peptides were synthesised (Australian Biobest Biotechnology Service) with a cysteine-glycine-glycine leader to enable orthogonal presentation on maleimide activated ELISA plates (Thermo Scientific). The peptides were reconstituted to 5 mg/mL using 1 x PBS, and 10 μg/mL of the peptides were captured onto the plates as per manufacturer’s instructions. A blocking step with M-PBS was included after inactivating excess maleimide groups using a cysteine solution. A direct ELISA protocol as outlined above was then performed using GUS2 and each of the serotype-specific antibodies at a concentration of 10 μg/mL.

Next we investigated the ability of Gus2 to bind in tandem with the serotype-specific mAbs in a sandwich ELISA format. 200 μL of 3 μg/mL Gus2 was immobilized on ELISA plate wells overnight at 4°C. Washes were performed between each step using PBS-T. Free binding surfaces were blocked using M-PBS. DENV rNS1 from each serotype was diluted to 3 μg/mL in M-PBS and 200 μL was added to relevant wells. Each of the reformatted serotype-specific mAbs, were conjugated to HRP (Thermo Scientific), and used to detect NS1 binding.

### Sandwich ELISA—Effect of antibody orientation on sensitivity

To determine the best orientation for the Sandwich ELISA with respect to assay sensitivity, the serotype-specific mAbs were used as either capture or detection reagents, and compared with a commercial Panbio^®^ Dengue Early ELISA assay, according to manufacturer instructions. 10 μg/mL of each serotype-specific antibody or 2.5 μg/mL of GUS-2 in PBS was used to coat ELISA plate wells overnight at 4°C. Washes were performed between each step using PBS-T. The wells were blocked with M-PBS after immobilisation. Following blocking, dilutions of DENV rNS1 in PBS from 1600 ng/mL to 0.05 ng/mL, were added to the wells and incubated for one hour. HRP-conjugated antibody from the Panbio^®^ Dengue Early ELISA Kit or HRP-conjugated serotype-specific antibodies at a concentration of 5 μg/mL were used for detection and an end-point absorbance signal was measured at 450 nm after stopping the reaction with 2 N sulphuric acid.

### Microsphere based serotyping assay

Carboxylated, modified microspheres with four different fluorescent profiles (FluoSpheres F8805, F8811, F8809 and F8807, Life Technologies) were functionalised using an adaptation of the method described by Sanjaya et al [23]. Briefly, the microspheres were washed three times and resuspended in 25 mM MES buffer, pH 5.5, 0.1% Pluronic F127. After activation of the carboxyl moiety using standard carbodiimide chemistry, the microspheres were rapidly washed twice by centrifugation and resuspended in PBS pH 7.4, 0.1% Pluronic F127. 10 mg BSA/mg microsphere was added and incubated for 2 h on an orbital shaker, then unbound BSA was removed by washing the microspheres four times by centrifugation and resuspension in PBS pH 7.4, 0.1% Pluronic F127. Azido-PEG_4_-NHS ester (Click Chemistry Tools, AZ103-100) was added to the microspheres and incubated for 30 minutes on an orbital shaker, then unreacted linkers were removed by centrifugation. In parallel, the serotype-specific mAbs were PEGylated using DBCO-PEG_4_-NHS ester (Click Chemistry Tools A134-100), using a 25-fold molar excess of linker to antibody for 30 min, then purified using a 40 kDa MWCO desalting column. The antibodies were then conjugated to the microspheres using alkyne-azide cycloaddition (25 ug of antibody / mg particles, incubated at 37°C for 3 h), with a different fluorescent microsphere for each serotype-specific mAb. Unbound antibodies were removed by centrifugation and the microspheres were suspended at 2 mg/mL in PBS, pH7.4, 0.1% Pluronic F127.

In order to demonstrate simultaneous discrimination of the NS1 serotypes, the conjugated microspheres were used as detection reagents in a sandwich immunoassay using immobilised Gus2 as the capture reagent. Human serum samples spiked with serial dilutions of NS1 were incubated for 1 h to enable efficient target capture. After a washing step, a mixture of the 4 types of conjugated, fluorescent microspheres was added to the wells. Removal of the unbound microspheres enabled fluorescent detection of the immobilised markers using a BMG ClarioStar microplate reader (excitation and emission filters for DENV-1: Ex360/50nm and Em415/10nm; DENV-2: Ex475/50nm and Em545/45nm; DENV-3: Ex525/35nm and Em580/25nm; DENV-4: Ex615/90nm and Em735/100nm).

## Results

Two phage libraries displaying scFv or Fab antibody fragments were interrogated for binders to DENV NS1. A subtractive biopanning strategy with three iterative rounds of biopanning for each DENV NS1 serotype and for each library was employed to isolate binders to immobilised, DENV rNS1. Binding to DENV rNS1 was confirmed by monoclonal phage ELISA, where 52 clones out of 90 screened from the DENV-1 pool were found to bind specifically to DENV-1 NS1. Similarly, for DENV-2, 8 out of 90; for DENV-3, 5 out of 90 and for DENV-4, 4 out of 90 were serotype specific. Other positive binders were also present but showed cross-reactivity with one or more other serotypes. After sequencing a selection of the positive clones, it was revealed that all of the DENV-1 clones were identical, all of the DENV-2 clones were identical and all of the DENV-3 clones were identical. There were two different sequences for the DENV-4 specific clones, but subsequent analysis showed that one of these clones was unable to bind natively sourced NS1. As such, four unique clones were taken forward for further characterisation: 9H2 (anti DENV-1), 4C11 (anti DENV-2), 7G11 (anti-DENV-3) and 6A5 (anti DENV-4).

**Fig 2:**
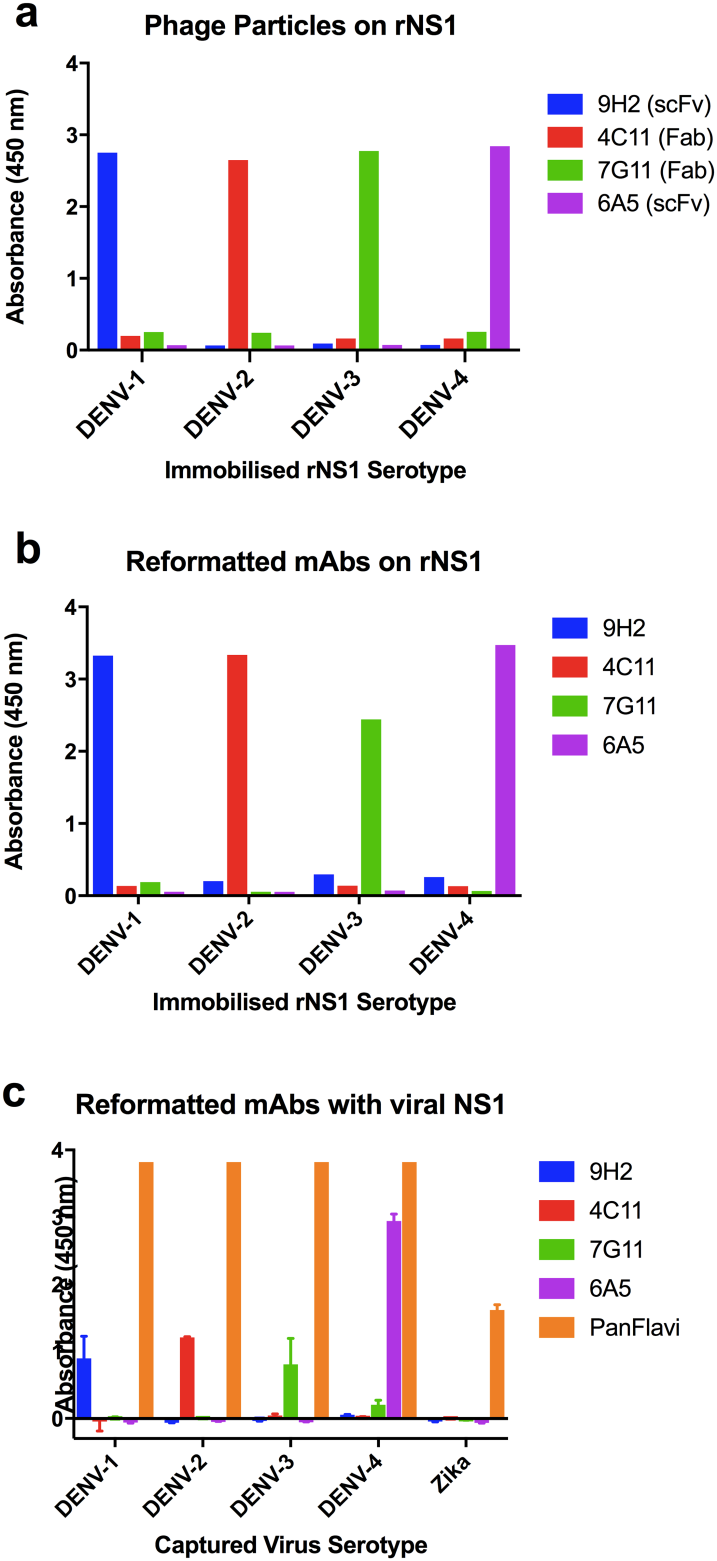
Reactivity of unique isolated phage clones and corresponding reformatted whole mAbs against rNS1 and native NS1 from all four DENV serotypes. **(a)** Monoclonal phage displaying antibody fragments from different libraries (either scFv or Fab) were added to immobilised recombinant NS1 (rNS1) and detected with a HRP-conjugated antibody against M13 filamentous phage. **(b)** Reformatted whole IgG were added to immobilised rNS1 and detected with a HRP-conjugated anti-human IgG antibody. **(c)** Flavivirus specific antibodies were immobilised and used to capture native NS1 from supernatants of Vero cells infected with DENV serotypes, Zika virus, or non-infected. Captured NS1 was detected using the serotype-specific reformatted mAbs or a flavivirus specific mAb.

The serotype-specific binding of these four phage clones to recombinant NS1 by direct ELISA is shown in Fig 2A.

Characterisation of the properties of isolated binders is important for determination of their potential utility in diagnostic assays. Equivalent characterisation of the isolated antibody fragment binders was facilitated by reformatting all fragments into fully assembled, bivalent IgG1. The reformatted IgG1 antibodies were assayed by direct ELISA, and serotype-specific recognition of recombinant antigen was conserved upon reformatting (Fig 2B).

**Fig 3:**
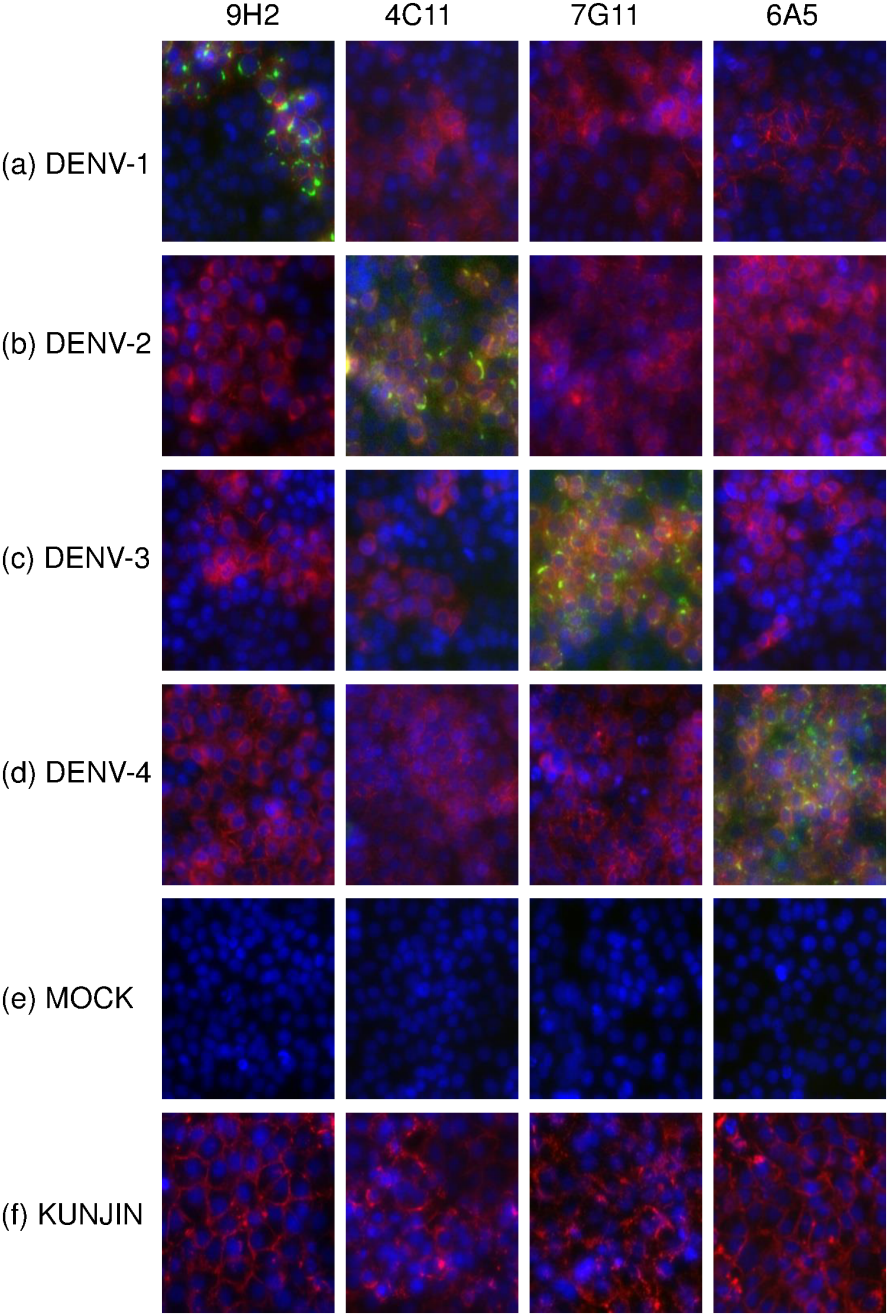
Immunofluorescence assay micrographs showing binding of serotype-specific anti DENV NS1 antibodies. Vero cells were infected with DENV-1 (a), DENV-2 (b), DENV-3 (c) DENV-4 (d), mock infection (e) or Kunjin virus (f). Viral infection is confirmed using an antibody to flavivirus envelope protein (4G2) stained with Anti-mouse IgG AlexaFluor546 (red), and serotype specific antibody binding (9H2, 4C11, 7G11 and 6A5) was detected with Anti-human IgG AlexaFluor488 (green). All wells also had a nuclear stain (Hoescht) (Blue) added to determine the relative localisation of binding.

One of the disadvantages of biopanning phage-displayed antibody fragment libraries on immobilised, recombinant antigen is that the immobilisation can sometimes lead to partial denaturation of some regions of the antigen. This can lead to isolation of binders to regions that are conformationally disparate to those present in well folded, native antigen. Furthermore, recombinant antigens often have tags that can be useful for purification but can unfortunately also lead to isolation of binders that target protein-tag junctions which are only part of the recombinant antigen. We sought to confirm that the serotype-specific IgG1 mAbs could bind to native DENV NS1. Serotype-specificity was confirmed on native NS1 using a sandwich ELISA to capture NS1 from the supernatant of DENV-, Zika- and mock-infected Vero cells, and no binding to Zika virus was observed (Fig 2C). Additionally, immunofluorescence assays using DENV-, mock- and Kunjin-infected Vero cells were performed to determine serotype-specific binding to membrane associated DENV NS1. The mock-infected Vero cells were used as a negative control while the Kunjin-infected Vero cells were used to test for cross-reactivity with another flaviviral NS1. All four serotype-specific antibodies maintained their serotype-specificity to native DENV NS1 as shown in Fig 3, and no cross reactivity with Kunjin virus was noted. Testing against a much larger panel of flaviviruses would be required to rule out cross-reactivity with all flaviviruses. Antibodies against DENV NS1 and the envelope protein showed peri-nuclear binding and diffuse cytoplasmic binding respectively.

The kinetic interactions of the serotype-specific antibodies with DENV rNS1 antigens were investigated using SPR; association and dissociation rates as well as the equilibrium dissociation constants (K_D_) are shown in Table 1. The weakest specific interaction was between 4C11 and DENV-2 NS1 (2.9 x 10^−7^ M), and the strongest between 6A5 and DENV-4 NS1 (3.5 x 10^−9^ M). No binding was detected between the serotype-specific antibodies and heterologous NS1 serotypes.

**Table 1:** Kinetics and affinities of antibody-antigen interactions. Kinetic data measured using SPR indicating association rate constants (k_a_) [M^-1^ s^-1^], dissociation rate constants (k_d_) [s^-1^] ± standard error as well as the equilibrium dissociation constants (K_D_) k_d_/k_a_ [M]. X denotes that no measurable interaction was detected by the assay.

	Analyte			
Antibody	DENV-1 NS1	DENV-2 NS1	DENV-3 NS1	DENV-4 NS1
**9H2**	k_a_ 4.7x10^4^ ± 150 k_d_ 6.7x10^-4^ ± 2.9x10^-6^ K_D_ 1.4x10^-8^	X	X	X
**4C11**	X	k_a_ 7.5x10^3^ ± 140 k_d_2.1x10^-3^ ± 1.5x10^-5^ K_D_ 2.9x10^-7^	X	X
**7G11**	X	X	k_a_ 4.5x10^4^ ± 89 k_d_ 7.1x10^-4^ ± 1.8x10^-6^ K_D_ 1.5x10^-8^	X
**6A5**	X	X	X	k_a_ 7.5x10^4^ ± 57 k_d_ 2.6 x 10^−4^ ± 2.2x10^-7^ K_D_ 3.5 x 10^−9^
**Gus2**	k_a_ 1.2x10^4^ ± 12 k_d_ 6.2 x 10^−5^ ± 7.9x10^-7^ K_D_ 5.4 x 10^−9^	k_a_ 1.1x10^5^ ± 140 k_d_ 1.9 x 10^−4^ ± 5.6x10^-7^ K_D_ 1.8 x 10^−9^	k_a_ 1.0x10^4^ ± 11 k_d_ 2.9 x 10^−4^ ± 7.5x10^-7^ K_D_ 2.8 x 10^−8^	k_a_ 1.1x10^4^ ± 17 k_d_ 2.0x10^-4^ ± 1.1x10^-6^ K_D_ 1.9 x 10^−8^

**Fig 4:**
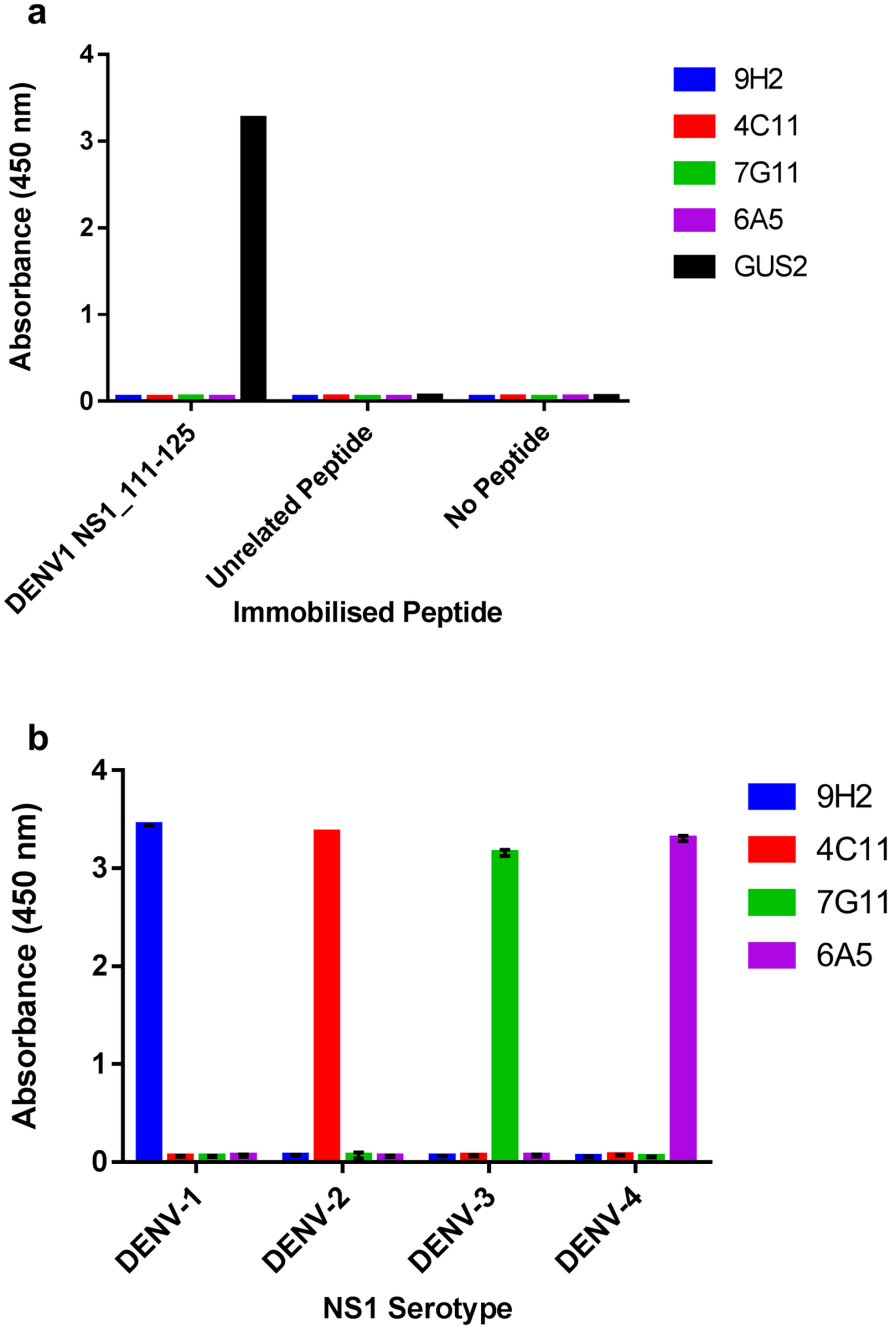
Investigation of serotype-specific mAb pairings with the DENV cross-reactive murine monoclonal Gus2. (a) Serotype-specific mAbs 9H2, 4C11, 7G11 and 6A5, and pan-reactive mAb Gus2, were tested for binding to an immobilised peptide consisting of DENV-1 NS1 amino acid residues 111–125, an unrelated peptide or no peptide. (b) Gus2 antibody was immobilised onto polystyrene plates and used to capture DENV rNS1. HRP-conjugated, serotype-specific antibodies (9H2, 4C11, 7G11 and 6A5) were then used for detection, showing the ability of these mAbs to sandwich with Gus2 for serotype-specific detection of NS1.

Sandwich ELISA relies on mutual exclusivity of the epitopes of capture and detection antibodies. The pan-reactive DENV NS1 antibody Gus2 was proposed for use as a sandwich ELISA pairing antibody for each of the serotype-specific mAbs. We confirmed that the serotype-specific mAbs do not bind to the same immunodominant peptide epitope as Gus2 (Fig 4A), and that each of the serotype-specific mAbs are able to pair with Gus2 in a sandwich ELISA, maintaining their serotype specificity (Fig 4B).

**Fig 5:**
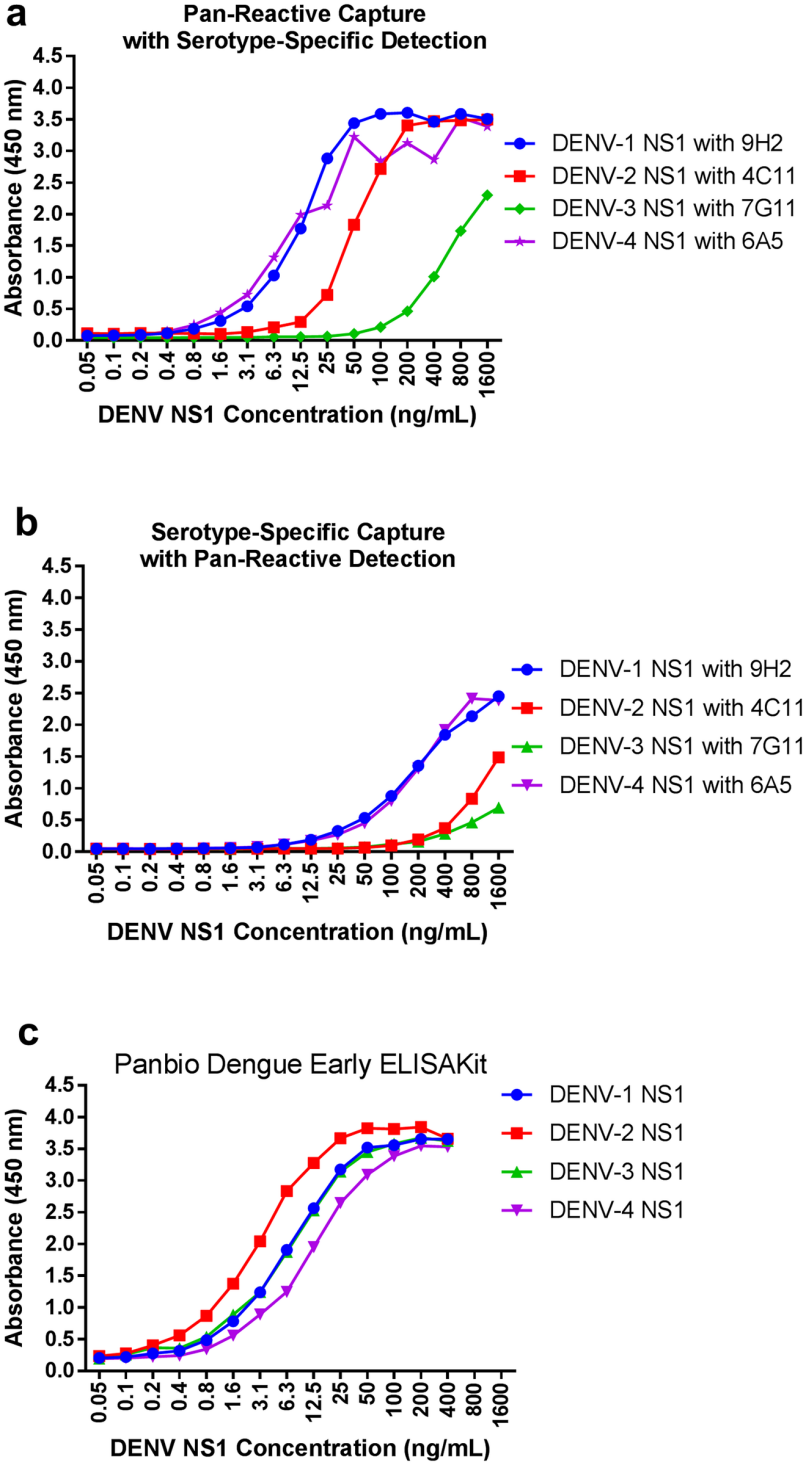
Investigation of effect of antibody orientation on sandwich ELISA sensitivity. Immobilised antibodies against DENV NS1 were used to capture dilutions of DENV rNS1. The serotype-specific antibodies were used either as the detecting antibody paired with pan-reactive Gus2 as the capture antibody (a), or as the capture antibodies with detection achieved used the detection antibody from the Panbio^®^ Dengue Early ELISA Kit (b). The results were compared with the complete Panbio^®^ Dengue Early ELISA Kit assay used as per the manufacturer's instructions (c). Background-subtracted data is shown indicating the mean ± SD.

We then sought to determine the best orientation for the sandwich ELISA, with respect to maximising sensitivity of the assay for detecting recombinant DENV1 NS1 in PBS. Sandwich ELISA was oriented either by using Gus2 as the capture antibody, with serotype-specific detection antibodies (Fig 5A), or in the opposite orientation (Fig 5B). These results were compared with the results of the non-serotyping commercial Panbio^®^ Dengue Early ELISA kit (Fig 5C).

The results show that the commercial assay format, which does not have serotyping capability, had greater sensitivity than either format of the serotyping assay when using serotypes DENV-2 and DENV-3. The inclusion of the novel antibodies however, adds a serotyping capability and, when used with the pan-reactive capture antibody Gus2, outperforms the commercial assay for detection of DENV-1 and DENV-4. The assay was less sensitive however, when the serotype specific antibodies were used as the capture antibody.

**Fig 6:**
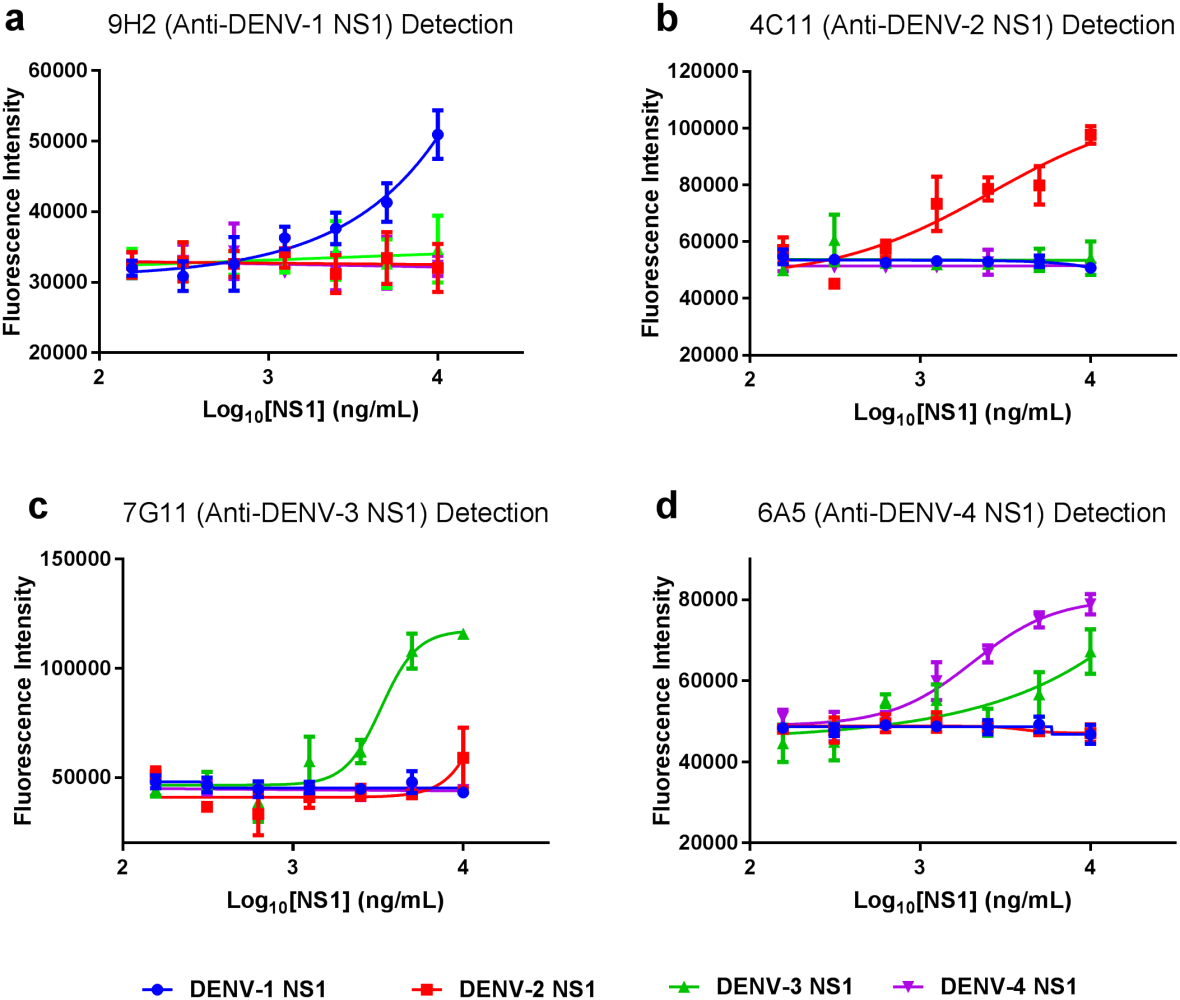
Proof of principle DENV serotyping assay in human serum using a multiplexed fluorescent microsphere based capture assay. Microplate wells were coated with Gus2 antibody, which was used to capture dilutions of NS1, separately for each DENV serotype, in human serum. Differentially fluorescent microspheres, conjugated with serotype-specific mAbs, were used to detect the capture of NS1. All four fluorescently labelled serotype-specific microspheres were added in each well. After washing, bound microspheres were detected using excitation and emission filters corresponding to each of the four microspheres (360nm/415nm for 9H2 Anti-DENV-1 (a), 475nm/548nm for 4C11 Anti-DENV-2 (b), 525nm/580nm for 7G11 Anti-DENV-3 (c), 615nm/735nm for 6A5 Anti-DENV-4 (d)).

Differentially-fluorescent microspheres, conjugated with the serotype-specific mAbs, allowed simultaneous discrimination of the NS1 serotypes in human serum. A dose-dependent curve was observed for each serotype with sensitivities between 10–100 ng/mL. Serotype specificity in this assay was excellent for DENV-1, -2 and -3, but DENV-4 NS1 detection showed some cross-reactivity with DENV-3 NS1 (Fig 6).

## Discussion

The use of antibodies in diagnostic assays has progressively grown over the years and the formats in which the antibodies are used have become diverse. Commonly, ELISA is used where antibodies specific for the target antigen are used for capture and subsequent colorimetric detection of the antigen. Newer diagnostic platforms are now beginning to replace these traditional approaches and include formats such as lateral flow, biosensor and lab-on-a-chip approaches which can offer advantages for both assay cost, simplicity and time to readout. Despite these advantages, they all fundamentally depend on the availability of antibodies with high affinity and selectivity for their target antigen.

This is particularly true in the case of dengue, where the co-circulation of four, serotypically distinct, dengue viruses together with a range of closely related flaviviruses places strict restraints on the utility of antibodies as diagnostic tools. Such restraints arise from the need to detect homologous regions of structurally related proteins, which presents a two-fold problem. Firstly, sequence divergence causes conformational differences of the structurally related biomarkers. The resultant differences may cause even the homologous regions to be displayed in conformations that may vary slightly or even grossly across the related proteins. This variation would lead to antibodies that bind two or more related proteins not being able to bind their target in an equivalent way. Secondly, from an immunological perspective, homologous regions can mount immunologically dominant responses where antibodies produced by B-cells preferentially bind that region. This is of particular relevance in DENV infections where secondary infection with a heterologous serotype leads to a targeted immunological response to the regions of shared homology. The resulting immune-complexed antigens may be rendered invisible to diagnostic assays as the relevant diagnostic epitopes are already occupied by endogenous antibodies. The variable specificities and sensitivities reported in multiple evaluations of commercial DENV NS1 detection assays illustrate these challenges [24–27].

In this study, we aimed to isolate serotype-specific DENV NS1 antibodies which could be used to improve the utility of DENV NS1 diagnostic assays. The use of phage display and a subtractive biopanning strategy directed the selection of binders towards serotype-specific epitopes, which is not feasible using immunisation strategies followed by conventional hybridoma technology. A previous report using phage display to isolate serotype-specific binders against NS1 did not incorporate a serotype-selective strategy, and isolation of serotype-specific binders was serendipitous and not successful for all four serotypes [28]. Serotype-specific antibodies have previously been isolated using immunisation and hybridoma technology, and these were used in a serotyping DENV NS1 ELISA [29–31]. Again, the approach was not a serotype-selective strategy so selection of serotype-specific mAbs required thorough screening amongst a majority of cross-reactive clones. An additional drawback with their assay was that the sandwich ELISA required a unique pair of serotype-specific antibodies for each DENV serotype which complicates the assay procedure.

We demonstrated that our phage library-derived serotype-specific antibodies can be paired with a single pan-reactive capture antibody. This compatibility enables a multiplexed diagnostic approach, and we showed that detection and serotyping can be performed in a single well, with the four detection antibodies conjugated to different fluorescent markers. Some serotype cross-reactivity with DENV-3 NS1 was observed when detecting DENV-4 NS1, indicating that the assay requires further optimisation. The observed cross-reactivity is likely due to sub-optimal optical settings on the fluorescence detector, rather than cross-reactivity of the mAbs. The settings for DENV-4 detection used excitation and emission filters with wide windows which overlap with the excitation and emission spectra of the microspheres used for DENV-3. A similar level of apparent cross-reactivity was observed previously using the same assay conditions with a different set of antibodies [32]. Further optimisation of the detection method is required before further assay development.Other multiplexed DENV serotyping assays based on RT-PCR have been described [33–35], but these rely on specialised equipment and therefore must be laboratory based. A serotyping immunoassay however could be readily translated to a rapid, point-of-care diagnostic test.

A disadvantage of isolating antibodies from naive phage libraries, is that the antibodies lack the *in vivo* affinity maturation aspect that occurs in natural humoral immune responses by somatic hypermutation [36]. This is evident in the moderate affinity of the serotype-specific antibodies isolated from the naive libraries in this study. Detection of DENV-2 and DENV-3 rNS1 in this work was markedly poorer than that of DENV-1 and DENV-4 rNS1 in the sandwich ELISA format, despite the interaction kinetics of the anti-DENV-3 antibody being similar to the anti-DENV-1 antibody. The limit of detection is governed by the cooperative kinetics of both the capture and detection antibodies, and both should be considered when attempting to improve the sensitivity of the assay.

Paramount to the affinity of the antibody for the antigen is the availability of sites on the antigen to which antibodies can bind. This is important in the background of a complex test matrix such as serum, especially during anamnestic infections where DENV NS1 may be sequestered in immune complexes [37]. Hypothetically, the non-immune driven, serotype-specific antibodies would still be able to participate in DENV NS1 capture or detection, if paired with a pan-reactive antibody against a non-immune epitope.

To conclude, interrogation of naive phage antibody libraries with a subtractive panning strategy has shown to be effective in isolating serotype-specific antibodies which could add a serotyping capability to existing NS1 assays, and we show that these have potential in a multiplexed immunoassay.
